# Change of Neural Connectivity of the Red Nucleus in Patients with Striatocapsular Hemorrhage: A Diffusion Tensor Tractography Study

**DOI:** 10.1155/2015/679815

**Published:** 2015-07-02

**Authors:** Sung Ho Jang, Hyeok Gyu Kwon

**Affiliations:** Department of Physical Medicine and Rehabilitation, College of Medicine, Yeungnam University, 317-1 Daemyung Dong, Namku, Daegu 705-717, Republic of Korea

## Abstract

The red nucleus (RN) is involved in motor control and it is known to have potential to compensate for injury of the corticospinal tract (CST). We investigated the change of connectivity of the RN (RNc) and its relation to motor function in patients with striatocapsular hemorrhage. Thirty-five chronic patients with striatocapsular hemorrhage were recruited. Motricity Index (MI), Modified Brunnstrom Classification (MBC), and Functional Ambulation Category (FAC) were measured for motor function. The probabilistic tractography method was used for evaluation of the RNc. Fractional anisotropy (FA), mean diffusivity (MD), and tract volume (TV) of the RNc were measured. FA and TV ratios of the RNc in patients with discontinuation of the affected CST were significantly higher than those of patients with preserved integrity of the CST in the affected hemisphere (*p* < 0.05). TV ratio of the RNc showed significant negative correlation with upper MI (weak correlation, *r* = −0.35), total MI (weak correlation, *r* = −0.34), and MBC (moderate correlation, *r* = −0.43), respectively (*p* < 0.05). We found that the neural structure of the RNc was relatively increased in the unaffected hemisphere compared with the affected hemisphere in patients with more severe injury of the CST.

## 1. Introduction 

The red nucleus (RN) is involved in motor control as the synaptic nucleus of the corticorubrospinal tract (CRST), consisting of the corticorubral tract (CRT) and the rubrospinal tract (RST) [1, 2]. The CRT mainly connects the motor regions of the cerebral cortex and cerebellum to mediate voluntary movement [1–4]. The RST is known to faciliate the flexor muscles of the upper extremity but is less important for the lower extremity [1, 2, 5, 6]. The CRST is closely related to the corticospinal tract (CST) in terms of anatomy, development, and function [1, 2, 5, 7–15]: (1) anatomy: the RST terminates in the lateral funiculus of the spinal cord, the same as the termination of the CST [1, 7–9], (2) development: the RST, which is initially involved in distal motor function, has been progressively substituted by the CST [13–15], and (3) function: the RST is important for motor control of the upper extremity, particularly the distal part of the upper extremity, like the CST [1, 2, 5, 7, 10–12, 16]. These suggest that the CRST has potential to compensate for motor function in cases of injury of the CST. Many animal studies have reported that the RN, including the CRST, is concerned with compensation for injury of the CST [13, 17–21]. However, due to the anatomical characteristics, direct investigation of the CRST in the live human brain is not easy, decussion at the lower midbrain and fewer axons than the CST [22]. Therefore, instead of direct research on the CRST, a few studies have investigated the RN and connectivity of the RN (RNc) in the human brain [3, 4, 19].

Change of neural connectivity is one of the evidences of compensation or recovery mechanism following brain injury [23, 24]. Previous animal studies have reported on neural change of the RN after brain injury, using various techniques such as electromyography, transcranial magnetic stimulation, and functional neuroimaging technique [6, 10–12, 17, 18, 20, 21]. However, these techniques have a common limitation in showing the change of a neural tract three-dimensionally. By contrast, diffusion tensor imaging (DTI) has a unique advantage in visualization and acquisition of quantitative data on the change of neural connectivity of a neural structure [25]. In particular, fiber tracking based on the multitensor model can consider both the dominant and nondominant orientation of diffusion in each voxel and shows how the regions of the brain are connected [25, 26]. Therefore, it has an advantage in investigating the change of neural connectivity of a neural structure. Many multitensor model DTI studies have reported on the neural connectivity of the neural structure in the human brain [3, 4, 26, 27]. However, no DTI study on change of RNc in stroke patients has been reported.

In the current study, using DTI, we attempted to investigate the change of the RNc and its relation to motor function in patients with striatocapsular hemorrhage.

## 2. Methods

### 2.1. Subjects

We recruited 35 consecutive patients (male: 22, female: 13; mean age: 52.7 ± 11.7 years; range: 32~79 years) who had been admitted for rehabilitation to the rehabilitation department of a university hospital for this study. Inclusion criteria for patients were as follows: (1) first ever intracerebral hemorrhage, (2) a hematoma located primarily in the striatocapsular regions, (3) DTI scan and clinical evaluation performed at chronic stage after onset (more than two months), and (4) no hydrocephalus, subarachnoid hemorrhage, or intraventricular hemorrhage. (5) Patients with apraxia or severe cognitive problems (Mini-Mental State Examination < 25) were excluded. This study was conducted retrospectively and the study protocol was approved by the Institutional Review Board of a university hospital.

### 2.2. Clinical Evaluation

Motor function of the affected upper and lower extremities was evaluated at the time of DTI scanning. The Motricity Index (MI), with a maximum score of 100, was used for measurement of motor function [28]. Upper MI included shoulder abduction, elbow flexion, and pinch grip, and lower MI included hip flexion and knee extension, and dorsiflexion. In addition, the Modified Brunnstrom Classification (MBC) and the Functional Ambulation Category (FAC) were used for measurement of function of the affected hand and gait function, respectively [29, 30]. Validity and reliability of the MI, MBC, and FAC are well established [29, 30]. Persons performing evaluations of clinical data were blinded to DTI data, and the person who performed analysis of DTI was also blinded to the clinical data.

### 2.3. Data Acquisition

DTI data were acquired at a mean of 16.9 ± 15.9 months from the onset of hemorrhage using a 6-channel head coil on 1.5 T Philips Gyroscan Intera (Philips Ltd., Best, Netherlands) with single-shot echo-planar imaging. For each of the 32 noncollinear diffusion sensitizing gradients, we acquired 67 contiguous slices parallel to the anterior commissure-posterior commissure line. Imaging parameters were as follows: acquisition matrix = 96 × 96; reconstructed to matrix = 128 × 128; field of view = 221 × 221 mm^2^; TR = 10,726 ms; TE = 76 ms; parallel imaging reduction factor (SENSE factor) = 2; EPI factor = 49; *b* = 1000 s/mm^2^; NEX = 1; and a slice thickness of 2.3 mm (acquired voxel size: 1.73 × 1.73 × 2.3 mm^3^).

### 2.4. Diffusion Tensor Tractography

The Oxford Centre for Functional Magnetic Resonance Imaging of the Brain (FMRIB) Software Library (FSL; http://fsl.fmrib.ox.ac.uk/fsl/fslwiki/) was used for analysis of diffusion-weighted imaging data. Affine multiscale two-dimensional registration was used for correction of head motion effect and image distortion due to the eddy current. Fiber tracking was performed using a probabilistic tractography method based on a multifiber model and applied in the current study utilizing tractography routines implemented in FMRIB diffusion (5000 streamline samples, 0.5 mm step lengths, and curvature thresholds = 0.2) [25, 26]. This fiber tracking technique by multifiber model calculated and generated 5000 streamline samples from seed region of interest (ROI) with consideration of both dominant and nondominant orientations of diffusion in each voxel and showed how the regions of the brain are connected [25, 26]. Therefore, it has an advantage in investigation of the neural connectivity of a neural structure. For the RNc, a seed ROI was placed on the isolated RN of the upper midbrain on the b0 map. Out of 5000 samples generated from the seed voxel, results for contact were visualized with the threshold of 100 streamlines through each voxel for analysis. In addition, we used the DTI-Studio software (CMRM, Johns Hopkins Medical Institute, Baltimore, MD, USA) based on the fiber assignment continuous tracking (FACT) algorithm to reconstruct the CST in the affected hemisphere to determine the integrity of the CST [31]. For reconstruction of the CST, two ROIs were given at the upper pons (anterior blue color) as seed ROI and pyramid (anterior blue color) as target ROI in the upper medulla on the color maps (color map: blue: superoinferior orientation, red: mediolateral orientation, and green: anteroposterior orientation) [32]. Fiber tracking was started at any seed voxel with a fractional anisotropy (FA) > 0.2 and ended at a voxel with a FA of <0.2 and a tract turning angle of <60 degrees. Values of FA, mean diffusivity (MD), and tract volume (TV) of the CST and RNc in both hemispheres were measured, and the ratios of FA, MD, and TV were calculated by dividing the value of the RNc into the unaffected hemisphere by the value of the RNc in the affected hemisphere.

**Figure 1:**
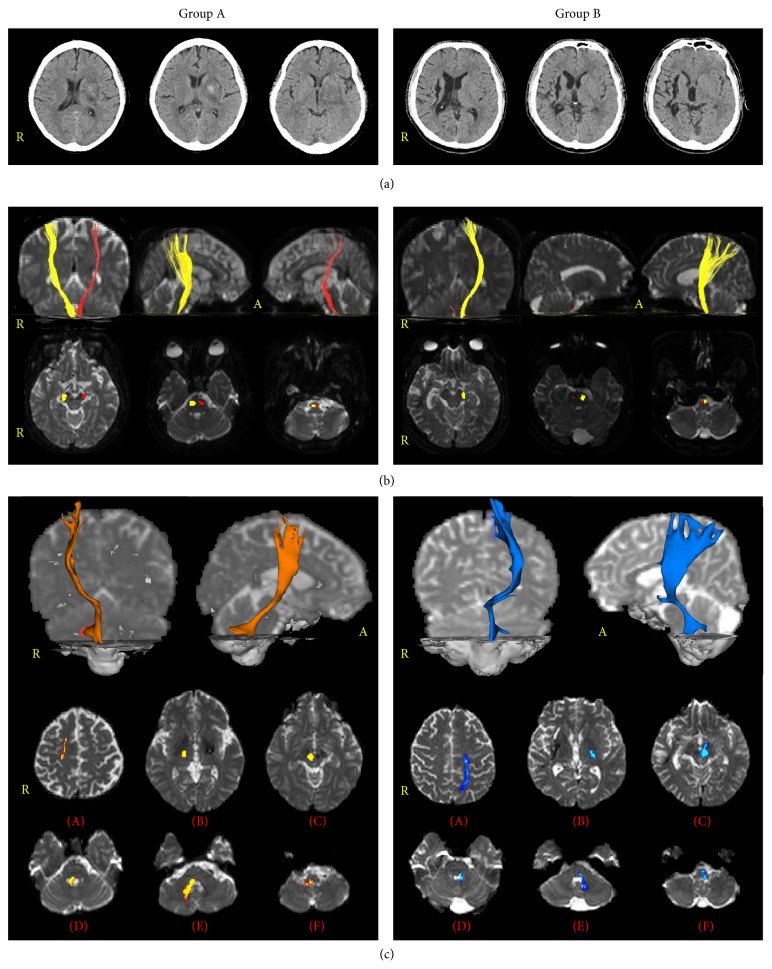
According to diffusion tensor tractography (DTT) findings of the corticospinal tracts (CST) based on integrity, all patients are classified according to two groups: group A, the CST in the affected hemisphere showing preservation of integrity from the cortex to the medulla, and group B, the CST in the affected hemisphere showing a discontinuation of integrity due to the lesion. (a) Brain CT images show the striatocapsular hemorrhage. (b) Results of DTT for the CST (yellow: unaffected hemisphere, red: affected hemisphere) and pathways of CST at the level of brainstem. CSTs descend the basis of pontine and terminate the pyramid of medulla. (c) Results of DTT for the neural connectivity between red nucleus and target brain regions ((A): cortex level, (B): internal capsule level, (C): upper midbrain level, (D): upper pons level, (E): lower pons level, and (F): medullar level).

According to diffusion tensor tractography (DTT) findings of the CST based on integrity, all patients were classified according to two groups: group A: the CST in the affected hemisphere showing preservation of integrity from the cortex to the medulla, and group B: the CST in the affected hemisphere showing a discontinuation of integrity due to the lesion (Figure 1).

### 2.5. Statistical Analysis

SPSS software (v.15.0, SPSS, Chicago, IL) was used for data analysis. An independent* t*-test was used for determination of variances in the DTI parameters of the CST, RNc, demographic data, and clinical data between group A and group B. Using Pearson and Spearman correlation, DTT parameters of the RNc in all patients were used in determination of correlation with motor function including the MI (Pearson), MBC (Spearman), and FAC (Spearman) and DTT parameters of the CST (Pearson) [33]. The significant level of the *p* value was set at 0.05.

## 3. Results

A summary of the demographic and clinical data for the patients is shown in Table 1. Among 35 patients, 13 patients (37.1%) belonged to group A and the remaining 22 patients (62.9%) to group B. Regarding the demographic data, no significant differences in demographic data were observed between groups A and B (*p* > 0.05). However, in the clinical data, significant differences in upper MI, lower MI, total MI, MBC, and FAC were observed between groups A and B, except for MMSE (*p* < 0.05).

**Figure 2:**
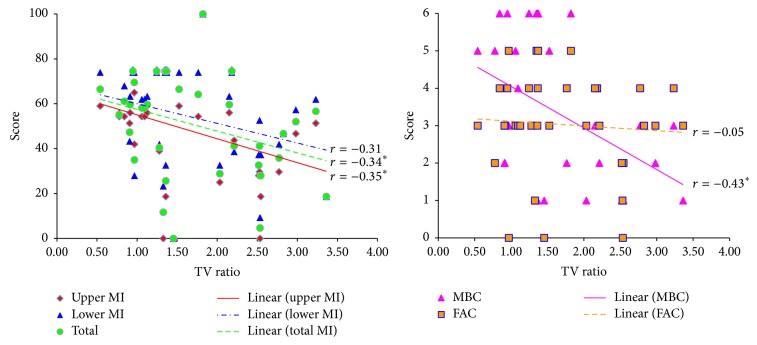
Correlations between motor function and diffusion tensor imaging parameters of connectivity of the red nucleus. MI: Motricity Index, TV: Tract volume, MBC: Modified Brunnstrom Classification, and FAC: Functional Ambulation Category. ^*∗*^ *p* < 0.05  .

Table 2 shows a summary of the DTI parameters of the RNc for the patients. Regarding the DTI parameters, no significant differences in FA, MD, and TV were observed between groups A and B (*p* > 0.05). In addition, motor function including upper MI, lower MI, total MI, MBC, and FAC did not show significant correlation with FA, MD, and TV (*p* > 0.05). By contrast, regarding the ratio of DTI parameters, FA and TV ratios of the RNc were significantly higher in group B than in group A (*p* < 0.05). However, no significant differences in MD ratio of the RNc were observed between group A and group B (*p* > 0.05). In addition, with regard to the correlations, TV ratio of the RNc showed significant negative correlation with upper MI (weak correlation, *r* = −0.35), total MI (weak correlation, *r* = −0.34), and MBC (moderate correlation, *r* = −0.43), respectively (*p* < 0.05) (Table 3 and Figure 2) [33]. However, TV ratio of the RNc did not show significant correlation with lower MI (*r* = −0.31) and FAC (*r* = −0.05), respectively (*p* > 0.05) [33]. In addition, FA and MD ratios of the RNc also showed no significant correlation with any motor functions (*p* > 0.05).

As for the correlation between the ratio of the RNc and the CST in both hemispheres, tract volume ratio of the RNc showed significant negative correlation with tract volume of the CST in the affected hemisphere (moderate correlation, *r* = −0.42) (*p* < 0.05) [33]. However, FA and MD ratios of the RNc did not reveal significant correlation with FA and MD of the CST in the affected hemisphere (*p* > 0.05). In addition, FA, MD, and tract volume ratios of the RNc did not reveal any significant correlation with FA, MD, and tract volume of the CST in the unaffected hemisphere (*p* > 0.05).

## 4. Discussion

In the current study, using DTI, we investigated change of the RNc and its relation to motor function of the affected extremities. Our results were as follows: (1) FA and TV ratios of the RNc were higher in group B than in group A and TV ratio of the RNc showed moderate negative correlation with tract volume of the CST in the affected hemisphere, and (2) TV ratio of the RNc showed negative correlation with upper MI, total MI, and MBC. In particular, MBC showed moderate negative correlation, whereas upper MI and total MI showed weak negative correlation; however, no significant correlation with lower MI and FAC was observed.

FA value indicates the degree of directionality of water diffusion and has a range of zero (completely isotropic diffusion) to one (completely anisotropic diffusion). It represents the white matter organization: in detail, the degree of directionality and integrity of white matter microstructures such as axon, myelin, and microtubule, and MD value indicates the magnitude of water diffusion [34]. In contrast, TV is determined by counting the number of voxels contained within a neural tract [35]. Therefore, an increment of FA and TV ratios of the RNc in the unaffected hemisphere in group B appeared to indicate increased relative directionality and voxel number of the RNc in the unaffected hemisphere compared with those of the affected hemisphere, respectively. In patients in group B, because the CST in the affected hemisphere showed a discontinuation of integrity due to the lesion, these results suggest that the neural structure of the RNc was relatively increased in the unaffected hemisphere compared with the affected hemisphere in patients with more severe injury of the CST. We believe that this appeared to result from compensation of more severe injury of the CST in patients of group B. In addition, this result appeared to be coincided with the result that tract volume ratio of the RNc showed moderate negative correlation with tract volume of the CST in the affected hemisphere.

Regarding the correlation with motor function of the affected extremities, only TV ratio showed negative correlation with mainly hand motor function (MBC) without correlation with lower motor function (FAC). These results indicate that the neural structure of the RNc was relatively increased in the unaffected hemisphere compared with the affected hemisphere in relation to poorer hand motor function of the affected side. Because the CST is mandatory for hand motor function, it appears that the relative increment of neural structure of the RNc in the unaffected hemisphere compared with the affected hemisphere may compensate for injury of the CST [36–40]. This result coincided with those of previous studies in that function of the RN including the CRST innervates mainly the upper extremity muscle [1, 2, 5, 7, 10–12, 16, 17]. Many animal studies have reported that the RN including the CRST was involved mainly in the motor function of the upper extremity, especially the distal movement of the upper extremity [10–12, 16, 17]. In 1999, Jarratt and Hyland reported that RN plays an important role in accurate hand movement in the rat using electrophysiological methods [10]. In 2001, van Kan and Mccurdy, who investigated two monkeys using electromyography with behavior tasks, explained that RN plays a key role in control of hand preshaping during reaching to grasp movement [12]. The next year, Küchler et al. (2002) demonstrated that distal forelimb muscles showed shorter latency than proximal muscles with 48 rats using electromyography [11]. They suggested that control of the distal forelimb muscle was mediated by the RST. By contrast, fewer studies have demonstrated involvement of the RN in lower motor function such as locomotion [6, 41]. However, our results did not show relation to lower motor function. We think that conduct of further studies on this topic should be encouraged.

The results described above demonstrate that the relative increment of neural structure of the RNc in the unaffected hemisphere compared with the affected hemisphere is also compatible with the results of previous studies reporting that the RN functionally connected the CST and it contributed to motor recovery as a compensation for injury of the CST [13–15, 17–21]. In 1968, two studies by Lawrence and Kuypers demonstrated that recovery of motor function was mediated by the RST in case of injury of the CST in monkeys [20, 21]. In 2000, Belhaj-Saïf and Cheney found that output effects to forearm muscles were mediated and reorganized by the RN after injury of the CST in monkeys [17]. During the same year, Z'Graggen et al. found that the CRT in the affected hemisphere crossed the midline and connected the contralateral RN in the rat with injury of the CST; as a result, they concluded that connectivity of the CRST substituted for injury of the CST [18]. Subsequently, in the human study, Yeo and Jang (2010) investigated the changes of DTI parameters of the RN at early stage (8–21 days after onset) in 49 patients with a corona radiata infarct, using DTI and transcranial magnetic stimulation [19]. They found that FA values of the RN in the affected hemisphere were higher than those of the RN in the unaffected hemisphere and FA value of RN in patients with more severe injury of the CST. As a result, they concluded that the RN in the affected hemisphere compensated for injury of the CST. Compared with these results, the results of the current study did not show increased activity of FA in the affected hemisphere; however, because the included patients, DTI timing from onset, and evaluation methods were different between these two studies, direct comparison is not possible.

## 5. Conclusion

In conclusion, we investigated the change of the RNc and its relation to the motor function of the affected extremities in patients with striatocapsular hemorrhage. It was found that the neural structure of the RNc was relatively increased in the unaffected hemisphere compared with the affected hemisphere in patients with more severe injury of the CST. In addition, the increased relative neural structure of the RNc in the unaffected hemisphere compared with the affected hemisphere was related to poorer hand motor function and injury of the CST in the affected hemisphere. We believe that our results might be a compensatory phenomenon for injury of the CST. Consequently, although the relative neural structure of the RNc was increased against injury of the CST in the affected hemisphere, it appeared not to be beneficial to motor recovery in patients with chronic striatocapsular hemorrhage. To the best of our knowledge, this is the first DTT study on change of the RNc in stroke patients. Therefore, we believe that the results of this study would be useful for clinicians in the field of neuroscience. However, several limitations of this study should be considered [42, 43]. First, we were obliged to investigate the RNc instead of the CRST because direct identification and investigation of the CRST in the human brain ARE difficult due to the limitation of the current technique. Second, multitensor model DTT can show false positive and negative findings due to fiber complexity or crossing fiber effect. Third, extrapyramidal tracts such as reticulospinal tract, vestibulospinal tract, and tectospinal tract can also influence motor function. Therefore, conduct of further studies to overcome the above-mentioned limitations should be encouraged.

## Figures and Tables

**Table 1:** Demographic and clinical data according to diffusion tensor imaging findings of the corticospinal tract for patients.

Variables	Group A	Group B	*p* value
Sex (male : female)	6 : 7	16 : 6	0.157
Mean age, year	56.2 (13.6)	50.5 (10.2)	0.169
Lesion side (right : left)	6 : 7	8 : 14	0.568
Duration from onset (months)	16.9 (18.0)	17.0 (14.9)	0.996
Mini-Mental State Examination	27.4 (2.6)	26.7 (2.9)	0.467
Motricity Index			
Upper extremity	68.1 (13.6)	35.3 (19.8)	0.001^*∗*^
Lower extremity	73.7 (9.1)	42.0 (18.9)	0.001^*∗*^
Total	70.9 (10.9)	38.6 (18.8)	0.001^*∗*^
Modified Brunnstrom Classification	5.15 (1.21)	2.14 (1.08)	0.001^*∗*^
Functional Ambulation Category	3.92 (0.76)	2.50 (1.37)	0.002^*∗*^

Values represent mean ± standard deviation.

^*∗*^ *p* < 0.05.

**Table 2:** Results of diffusion tensor imaging parameters of the connectivity of the red nucleus.

Hemisphere	Group A	Group B	*p* value
FA			
Affected	0.40 (0.03)	0.39 (0.04)	0.654
Unaffected	0.41 (0.02)	0.43 (0.03)	0.086
Ratio (unaffected/affected)	1.03 (0.06)	1.09 (0.08)	0.031^*∗*^
MD			
Affected	0.98 (0.09)	1.04 (0.10)	0.101
Unaffected	0.90 (0.05)	0.92 (0.08)	0.715
Ratio (unaffected/affected)	0.93 (0.08)	0.89 (0.10)	0.221
TV			
Affected	1338.1 (635.2)	1113.3 (606.2)	0.305
Unaffected	1604.1 (560.9)	1975.6 (772.8)	0.140
Ratio (unaffected/affected)	1.32 (0.44)	1.94 (0.85)	0.019^*∗*^

Values represent mean ± standard deviation, FA: fractional anisotropy, MD: mean diffusivity, and TV: tract volume.

^*∗*^ *p* < 0.05.

**Table 3:** Correlations of the ratio of diffusion tensor imaging parameters of the connectivity of the red nucleus with motor function and diffusion tensor imaging parameters of corticospinal tract.

	FA ratio	MD ratio	TV ratio
MI			
Upper extremity	*r* = − 0.10	*r* = 0.09	*r* = −0.35^*∗*^
Lower extremity	*r* = − 0.14	*r* = 0.10	*r* = − 0.31
Total	*r* = − 0.12	*r* = 0.09	*r* = −0.34^*∗*^
MBC	*r* = − 0.19	*r* = 0.22	*r* = −0.43^*∗*^
FAC	*r* = − 0.01	*r* = − 0.06	*r* = − 0.05
DTT parameter			
CST (affected side)	*r* = − 0.27	*r* = − 0.05	*r* = −0.42^*∗*^
CST (unaffected side)	*r* = − 0.27	*r* = 0.25	*r* = − 0.06

Pearson and Spearman correlation for Motricity Index, Modified Brunnstrom Classification, Functional Ambulation Category, and diffusion tensor tractography parameter. FA: fractional anisotropy, MD: mean diffusivity, TV: tract volume, MI: Motricity Index, MBC: Modified Brunnstrom Classification, FAC: Functional Ambulation Category, DTT: diffusion tensor tractography, and CST: corticospinal tract.

^*∗*^ *p* < 0.05.
